# Structural Insight of the Full-Length Ros Protein: A Prototype of the Prokaryotic Zinc-Finger Family

**DOI:** 10.1038/s41598-020-66204-5

**Published:** 2020-06-09

**Authors:** Gianluca D’Abrosca, Antonella Paladino, Ilaria Baglivo, Luigi Russo, Marica Sassano, Rinaldo Grazioso, Rosa Iacovino, Luciano Pirone, Emilia Maria Pedone, Paolo Vincenzo Pedone, Carla Isernia, Roberto Fattorusso, Gaetano Malgieri

**Affiliations:** 1 Department of Environmental, Biological and Pharmaceutical Sciences and Technologies, University of Campania “Luigi Vanvitelli”, via Vivaldi, 43, 81100 Caserta, Italy; 2 SCITEC-CNR, via Mario Bianco 9, 20131 Milano, Italy; 3 Institute of Biostructures and Bioimaging - CNR, Via Mezzocannone 16, 80134 Naples, Italy

**Keywords:** Protein structure predictions, Molecular modelling, NMR spectroscopy

## Abstract

Ros/MucR is a widespread family of bacterial zinc-finger (ZF) containing proteins that integrate multiple functions such as virulence, symbiosis and/or cell cycle transcription. NMR solution structure of Ros DNA-binding domain (region 56–142, i.e. Ros87) has been solved by our group and shows that the prokaryotic ZF domain shows interesting structural and functional features that differentiate it from its eukaryotic counterpart as it folds in a significantly larger zinc-binding globular domain. We have recently proposed a novel functional model for this family of proteins suggesting that they may act as H-NS-‘like’ gene silencers. Indeed, the N-terminal region of this family of proteins appears to be responsible for the formation of functional oligomers. No structural characterization of the Ros N-terminal domain (region 1–55) is available to date, mainly because of serious solubility problems of the full-length protein. Here we report the first structural characterization of the N-terminal domain of the prokaryotic ZF family examining by means of MD and NMR the structural preferences of the full-length Ros protein from *Agrobacterium tumefaciens*.

## Introduction

Ros/MucR is an important family of prokaryotic proteins that includes Ros from *Agrobacterium tumefaciens*^1–3^, RosR from *Rhizobium etli*^4^, RosR from *Rhizobium leguminosarum*^5,6^, MucR from *Brucella* spp^7–9^, MucR from *Sinorhizobium meliloti*^10,11^, MucR from *Sinorhizobium fredii*^12^; they all regulate genes required for the interaction of these bacteria with plants or animals^11,13–20^. Furthermore, MucR1 and MucR2 in *Caulobacter crescentus* have been recently shown to participate in the coordination of the systematic expression of the cell cycle genes^21,22^.

We have recently proposed a novel functional model for the prokaryotic zinc-finger (ZF) proteins belonging to the Ros/MucR family:^23–25^ they act as H-NS-‘like’ gene silencers^26,27^ by binding low consensus AT rich regions in DNA rather than functioning like their eukaryotic counterparts that mainly act as DNA sequence-specific transcriptional regulators. We have also demonstrated how MucR oligomerization is a key feature for its proper gene regulatory function in *Brucella*.

From an evolutionary standpoint, the prokaryotic ZF has been proposed as the ancestral domain from which the eukaryotic Cys_2_His_2_ ZF domain has evolved^28,29^. Structural and functional similarities and differences between the prokaryotic and the eukaryotic ZF have been largely documented^3,29–37^: the two domains are similar in the tetrahedral coordination of a structural zinc ion^38^ and in the existence of a ββα topology surrounding it while they differ in the fact that the prokaryotic ZF shows a second α-helix and a larger hydrophobic core. Moreover, this latter domain can bind the structural metal ion with a variable set of coordinating residues or even overcome the structural metal ion requirement to properly fold and function^39–43^. NMR solution structure of Ros DNA-binding domain (region 56–142, i.e. Ros87) has been solved more than a decade ago^30^. Ros87 globular domain presents a βββαα topology, where the zinc ion is fully coordinated and supports the structural organization of the protein fold. No structural characterization of the Ros N-terminal domain (region 1–55) is available to date, mainly because of serious solubility problems of the full-length protein. Nonetheless, as formerly reported, the N-terminal domain of this family of proteins appears to be responsible for the formation of higher-order oligomers^23–25^. Structural predictions of the *Brucella* MucR based on the structural propensity of the amino acid sequence, report two α-helices as characterizing the N-terminal region of this family of proteins^24^. A number of deletions and single residue mutants have been designed to prove the involvement of these helices in the formation of the quaternary structure necessary for MucR function. Here, we report the first structural characterization of the N-terminal oligomerization domain of the prokaryotic ZF family investigating the structural preferences of the full-length Ros protein from *Agrobacterium tumefaciens* by means of Molecular Dynamics studies and Nuclear Magnetic Resonance experiments.

## Results

### Full-length Ros models

**Figure 1:**
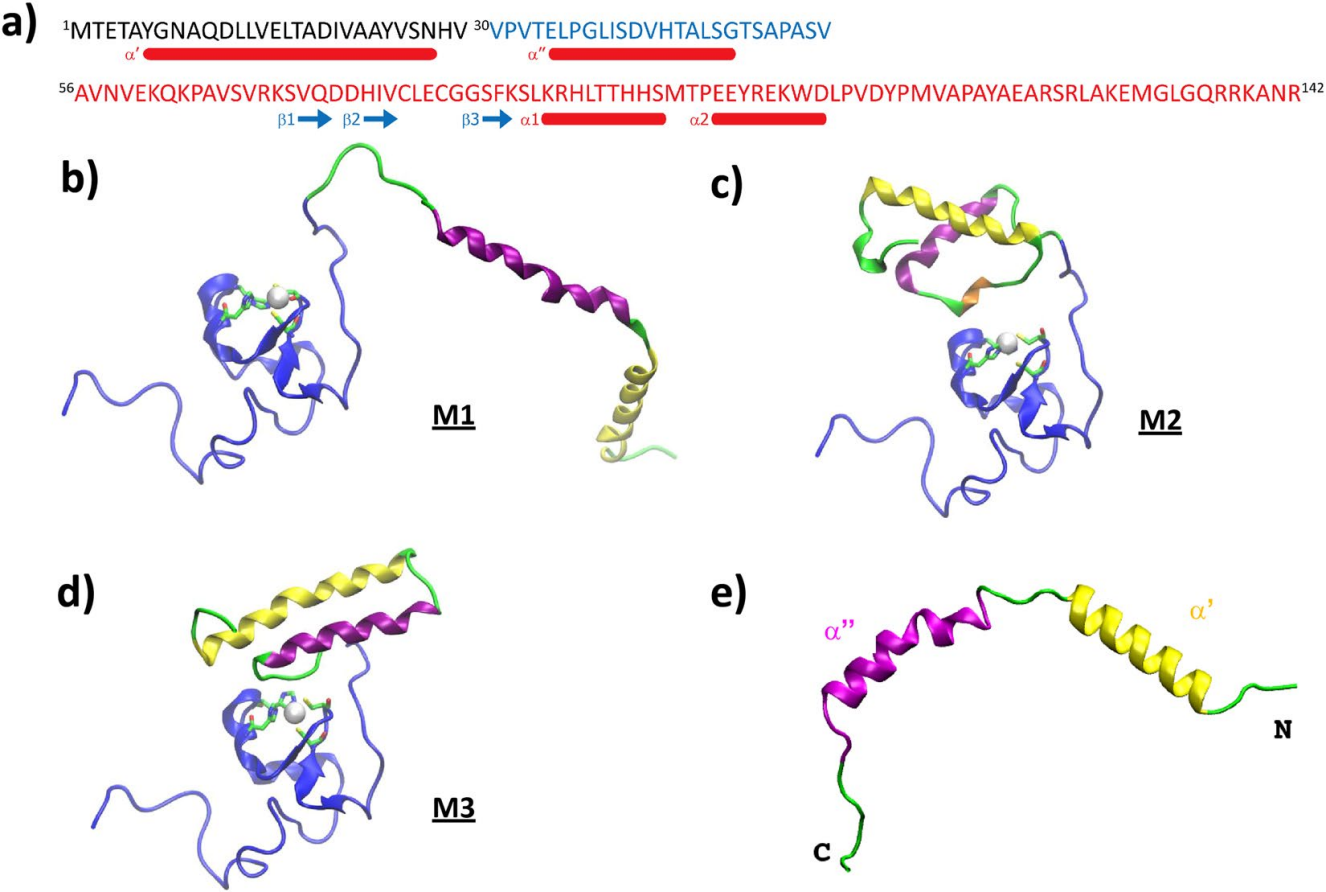
(**a**) Ros sequence. Full-length Ros protein primary sequence: in red is highlighted the C-terminal globular domain (amino acids 56–142, Ros87) and in blue the additional region of del29-Ros (amino acids 30–55). The secondary structure elements of the NMR structure (β1 -amino acids 72 and 73-, β2 -from 76 to 78-, β3–85 and 86-, α1 -from 89 to 97- and α2 -from 101 to 108-) and of one of the proposed models (a′ -amino acids 6–27- and a′′ -amino acids 34–48- of M3) are represented under their corresponding sequences. **(b-e)** 3D structures. Structural models of the full-length Ros (amino acids 1–142; a-c) and of the N-term domain (amino acids 1–55; d) are shown in cartoon: a) M1, b) M2 and c) M3. Helices of the N-domain are rendered in yellow (α′), purple (α′′) and orange (α′′′ in M2). See main text. In C-domain (blue) zinc-coordinating residues are indicated in sticks and Zn(II) is a white sphere.

The full-length Ros protein structure (the sequence is shown in Fig. 1a) was built using two different strategies: by means of comparative modelling^44^ and by evaluating amino acid intrinsic secondary structure propensities^45^. In the first approach, we produced homology models of protein domains obtained by detecting PDB homologs (or fragments with similar local sequences) used as templates^46^ to build native-like structures using Rosetta algorithm^47^. In the second, secondary structure propensities of the N-term amino acids were used to manually model and refine N-domain (see Methods). Such domain was then joined to the NMR solution structure of the C-terminal domain (Ros87 - 2JSP) using Maestro software package^48^. Next, we evaluated and selected a very extended 2-helices N-term domain structure (Fig. 1b) and the first two best-ranked N-term domain structural predictions (Fig. 1c,d), ending up with 3 different full-length (142 residues) protein models where the predicted N-term domain is linked to the high-resolution NMR C-term domain (Ros87 structure).

Figure 1 shows the starting models of the full-length protein where the C-term part (Ros87) typically adopts the βββαα fold, accommodating the zinc-binding region.

Two out of the three full-length models (M1, M3) present 2 long α-helices characterizing the N-terminal domain: namely α′ (Tyr6-Ala23), α′′ (Asn27-Thr45) for M1 and α′ (Tyr6-Asn27), α′′ (Glu34-Gly48) for M3 linked to Ros87. The same domain for the M2 model is made by 3 shorter helices, namely α′ (Gln10-Ser26), α′′ (Val32-Glu34) and α′′′ (Gly37-Glu48).

To further evaluate the intrinsic folding propensities of the N-terminal domain (amino acids 1–55) and its effect on the structural rearrangement of the full-length Ros, we carried out additional molecular dynamics simulations of the isolated N-domain (Figs. 1e and SI1b), composed by the two fully extended helices α′ and α′′.

It is worth underlining that the use of extensive MD simulations and several starting models are planned to overcome initial approximations due to the lack of structural data.

### Cluster analysis

**Figure 2:**
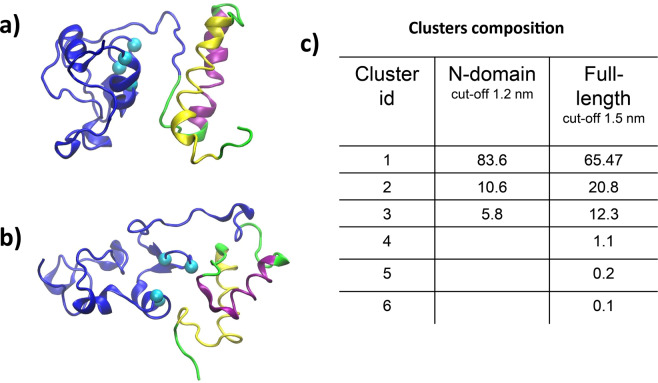
Cluster analysis. Ros structure upon least square fit on the N-terminal domain (**a**) and full-length protein (**b**). Centroids of the corresponding first cluster are displayed and rendered in cartoon: C-domain is colored blue, N-domain is divided in two main helices, α′ (Y6-N27) and α′′ (E34-G48) for clarity (see main text). Cyan balls localize the c-alpha of the zinc coordination sphere (C79, C82, H92, H97). (**c**) Details of cluster analysis given in terms of percentage of cluster composition (see Methods).

MD simulations on the obtained models were run as described in the materials and methods section and the conformational sampling of the full-length Ros was investigated in terms of cluster analysis. The integrity of the simulations and the stability of the modelled structures were evaluated by means of atomic position deviations (Figure SI2). The full meta-trajectory (600 ns) was analysed in the attempt to classify local structural features and corresponding Ros conformations. Cluster analysis is performed on the C-alpha atoms of either the full-length protein after least square fitting on the full-length (142 amino acids) or the N-term domain (55 amino acids), with an RMSD cut-off of 1.5 and 1.2 nm, respectively (RMSD cut-offs are the average root mean square deviations along the meta-trajectory). Correspondent Ros conformations are extracted for each cluster centroid for both procedures. Figure 2 displays the population coverage per cluster (i.e. conformation) computed along the meta-trajectory.

**Figure 3:**
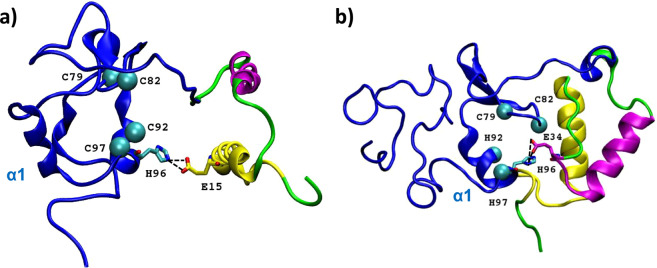
Key ionic interactions between N- and C-terminal domains. First cluster representatives upon least square fit on the N-terminal (**a**) and full-length (**b**) Ros. E15 from α′ helix (yellow), E34 from α′′ helix (magenta) and H96 from the C-domain (blue) involved in a salt bridge interaction are displayed in stick.

Clustering studies underline the high conformational variability of the full-length protein that converges in a preferential structural arrangement: conformations where the N-term and the C-term domains come together turns out to be more stable/visited (Fig. 2a,b). Indeed, in the most representative structures, the two domains are stabilized in a globular shape by establishing strong electrostatic interactions (Fig. 3).

On the other side, only a small population is made by a very disordered and extended N-domain, namely accounting for ~20% (cluster #2 in the full-length analysis) and ~10% (cluster #2 in the N-domain analysis) of the entire trajectory in the table reported in Fig. 2c.

In the clustering obtained by fitting onto the N-terminal domain, more than 83% of the conformational ensemble coincides with a protein structure characterized by a highly structured N-terminal domain made by α′ (Tyr6-Asn27) and α′′ (Glu34-Gly48) helices, closely resembling M3 model in Fig. 1d.

Importantly, both representative Ros structures of the first clusters (Fig. 2a,b) exhibit a conserved secondary structure element at the N-term level. Specifically, in most of the conformations, helix α′′ represents a common lasting motif, whereas the first region of the N-term, from amino acid 1 to amino acid 33, displays a larger flexibility sampling different helical arrangements. The evolution of secondary structure elements during the trajectory confirms a net tendency by the N-terminal domain to shape into helical arrangements. In Fig. SI1a, large blue stripes in the simulation window between 200 ns and 600 ns of the trajectory indicate a very stable inclination to form 2 long α-helices. It is worth noticing that in the first part of the simulation time, 0–200 ns (corresponding to the fully extended N-terminal helices, M1), the structural rearrangement of the N-term domain appears highly fluctuating albeit capable of adopting alternative helical structures (3- and 5-helices).

In this framework, comparison with the simulations of the N-domain fragment (amino acids 1–55) is useful to underscore the key role played by the C-term domain (Ros87) in stabilizing the 2-helices of the N-terminal domain. In Fig. SI1b, the N-terminal domain exhibits stretches of irregular helical rearrangement, with a long lasting yet variable α′′ (3- and 5-helices). Furthermore, the main representative structure (data not shown) of the MD simulation carried out on the N-terminal domain (over 400 ns of simulation time) adopts a preferential globular rearrangement, whereby helix α′′ splits into two smaller helices (magenta in Fig. SI3) by rotating and exposing its hydrophobic face to α′ helix, thus optimizing its solvent exposition.

**Figure 4:**
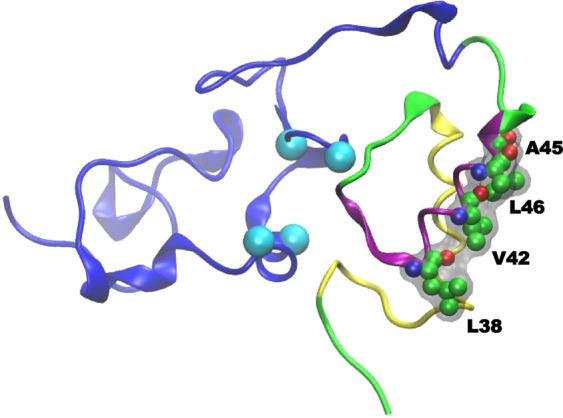
Hydrophobic patch. Amino acids of the N-domain α′′ helix forming a continuous hydrophobic surface are labeled and evidenced in CPK. Protein structure is shown in cartoon: C-term domain (Ros87) is blue, N-term domain is divided in two helices, α′ (Y6-N27) and α′′ (E34-G48) for clarity. Cyan balls localize the c-alpha of the zinc coordination sphere (C79, C82, H92, H97).

In this framework, it is worth underlining that in the full-length Ros (cluster centroid, Fig. 2b) α′′ helix shows a hydrophobic surface (Fig. 4) made by amino acids Leu38, Val42, Ala45 and Leu46 that may account for the mentioned aggregation properties^24^.

### Intra-molecular interactions

The C-term domain (Ros87) appears to have a key role in stabilizing the 2-helices (α′ and α′′) of the N-terminal domain. For this reason, we have focused our attention on the interactions at the interface between the two domains. The number of hydrogen bonds between the two subunits (amino acids 1–55 and 56–142) ranges from 1 to 7 along the entire meta-trajectory and key salt bridges are established. Among these, interesting ionic interactions take place occasionally between the α′′-helix residues (Glu34 and Asp41) and amino acids from the loop region (amino acids 61–70) preceding the C-term domain βββ structure (i.e. Asp41-Lys63, present for the 10% of simulation time). Full-length Ros globular state might be primed and favoured by stable bonds occurring between the two domains and stabilizing their reciprocal orientation. In Fig. 3, ionic interactions corresponding to the most representative structures are shown: His96 (shielding Zn(II) coordination site in Ros87 structure) is alternatively bridged to α′ and α′′ helices of the N-term domain.

Notably, from Fig. 2 the most populated conformations show a fairly globular shape where the compact C-term structure (Ros87) becomes interfaced with the N-term domain. Nevertheless, in this structural rearrangement, the new interaction surface does not directly involve the zinc-binding site, and more importantly, C-term domain α1 is kept accessible in all conformations, consistently with its role in DNA-binding already discussed^49^.

### del29-Ros contains Ros87 structure and preserves its features

**Figure 5:**
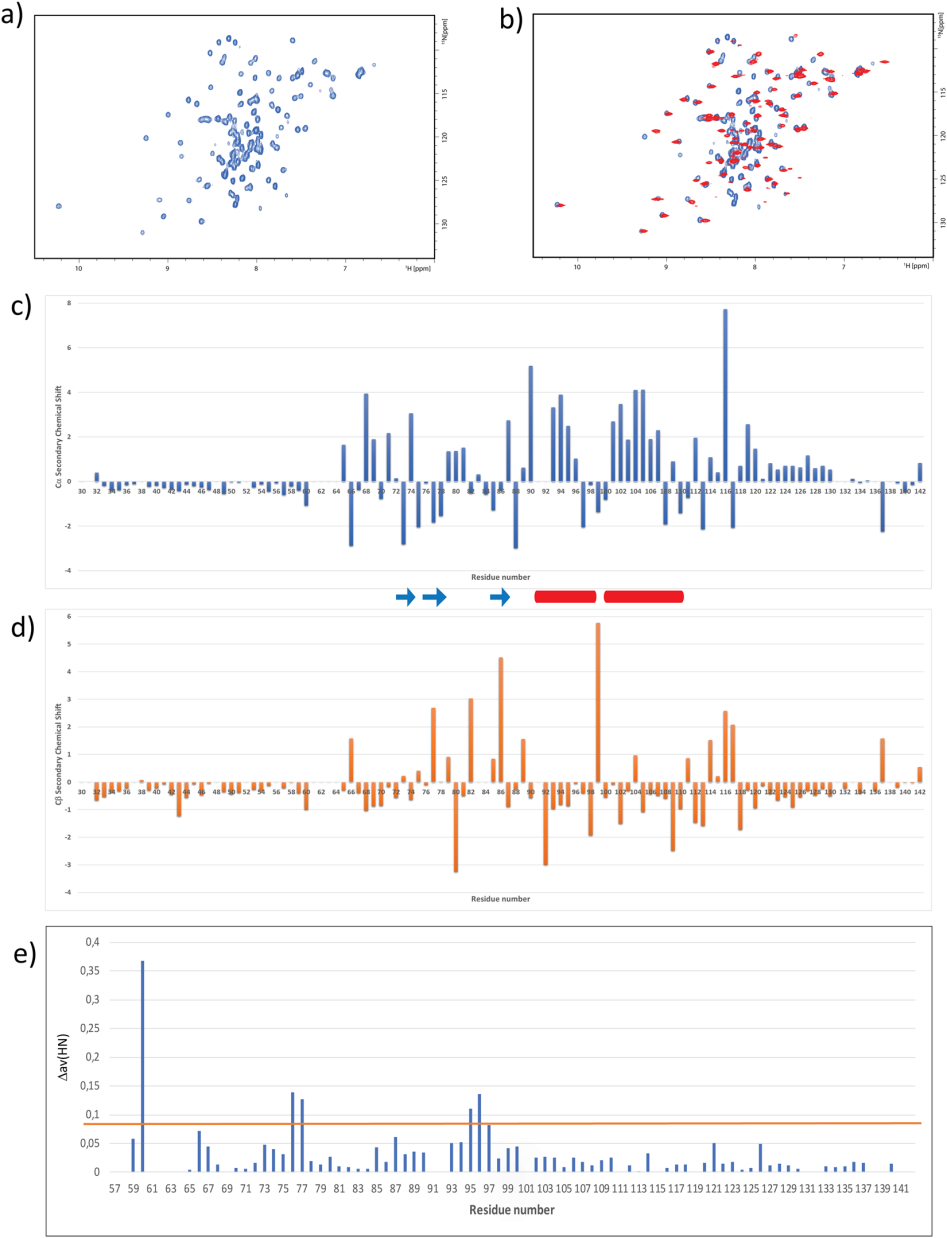
NMR analysis. (**a**) ^1^H-^15^N HSQC spectrum of del29-Ros. (**b**) Overlay of del29-Ros (in blue) and Ros87 (in red) ^1^H-^15^N HSQC spectra. (**c**,**d**) Secondary Chemical Shift for Cα (**c**) and Cβ (**d**) of del29-Ros; the secondary structure elements of the C-term domain (Ros87) are also reported. (**d**) Chemical Shift Perturbation of del29-Ros against Ros87; the orange line represents the mean value plus the standard deviation.

As stated in the introduction section, while Ros87, the minimum DNA binding region of the protein obtained by deletion of the first 55 amino acids, has been largely characterized, the structural characterization of the full-length Ros protein has been always impaired by solubility problems. For these reasons, we have resorted to carry out a structural analysis of del29-Ros by means of NMR spectroscopy. del29-Ros is the soluble deletion mutant designed by excluding the first predicted helical portion of the protein (α′). The NMR characterization commenced with the analysis of the DOSY experiment recorded for this deletion mutant. This experiment gave a diffusion coefficient (D_t_) of 1.16 (±0.11) *10^−10^ m^2^sec^−1^, which is quite comparable to the same value obtained for Ros87^30^ and demonstrates the monomeric form of this mutant also at the NMR concentration used^50,51^. LS experiment confirmed a monomeric structure for del29-Ros (Fig. SI4). Accordingly, chemical cross-linking experiments with dimethyl adipimidate (DMA) show how del29-Ros is capable of forming only an irrelevant amount of oligomers (Fig. SI5). Figure 5a reports del29-Ros ^1^H-^15^N HSQC spectrum which shows that this mutant, adopts a well-defined native structure in solution with considerable tertiary interactions. The spectrum is characterized by a considerable part of resonances nicely dispersed within a chemical shift range of ∼3 ppm in the ^1^H and ∼24 ppm in the ^15^N dimension. This aspect distinctly outlines the presence of β-strands, as this secondary structure commonly gives a good dispersion of the NMR signals. The centre of the spectrum contains less dispersed signals, likely resonances from helical structures known to produce a minor degree of dispersion.

Figure 5b reports the superposition of the ^1^H-^15^N HSQC spectrum recorded for Ros87 with the same spectrum acquired in the same conditions of this latter mutant. It is clear that Ros87 domain, if one excludes minor local differences, is essentially contained within the structure of del29-Ros.

In order to complement the available structural data, resonance assignments for H_N_, ^15^N, Cα, CO and Cβ nuclei were obtained for del29-Ros utilizing inter-residue connectivities detected in standard triple-resonance experiments. The obtained chemical shifts are reported in Table SI1. The examination of the ‘secondary shifts’ (deviations from random coil values) of the Cα, Hα, CO and Cβ resonances, all of which have been empirically correlated to secondary structure propensities^52^, allowed the identification of del29-Ros secondary structure elements (Fig. 5c,d). Helical structures give positive Cα and CO and negative Hα and Cβ secondary shifts, while β-strand structures give negative Cα and CO and positive Hα and Cβ secondary shifts. As shown by the figure, the helical and β-strand elements predicted on the base of the assigned chemical shifts are in good agreement with those observed in Ros87 NMR structure (the C-terminal portion of del29-Ros) while the N-terminal part (residues 30–55) does not appear to fold in any predominant secondary structure element and is, in contrast, structurally disordered. While on the one side, the removal of the region 1–29 does not impair the structural organization of the C-term Ros87, on the other it is associated to a low order structuration of the fragment 30–55, discordant with a helical arrangement. Consistent with our model in which the two helices are packed together to form hydrophobic interactions that appear to stabilize both helical structures, the removal of the first helical region does not allow the formation of also the second helical structure.

### del29-Ros N-term influences on Ros87 domain

We have compared the ^1^H-^15^N HSQC spectra of Ros87 and of del29-Ros (Fig. 5b) and mapped the found differences (Fig. 5e) on Ros87 NMR structure in Fig. SI6.

Significant chemical shift perturbations within the Ros87 region are mainly sited in three distant portions of the protein: the 56–60 portion, His76-Ile77 and Thr95-His96; the latter two constitute respectively the first β-strand and the last turns of α1 (helix 1 in Ros87).

While it is quite plausible that the removal of del29-Ros N-terminal region to obtain Ros87 perturbs the chemical environment of the contiguous 56–64 portion, the structural perturbation of the C-terminal domain requests a supplementary molecular elucidation. The CSI analysis has shown no significant differences in secondary structure content and distribution of the two proteins. According to the simulation data, a transient interaction between the hydrophobic N-terminal part of del29-Ros and His96 could support this observation, allowing del29-Ros to populate, even at a smaller extent, a more compact structure.

## Discussion

The ability to form oligomers is emerging as a key structural feature for the regulatory function played by the Ros/MucR family of prokaryotic ZF containing proteins. We have previously demonstrated that the deletion of the first 55 amino acids (Ros87-the minimal DNA binding motif) gives soluble monomeric protein capable of binding DNA, while the full-length protein has been showed capable of forming high order oligomers^24^.

We have also demonstrated that a monomeric MucR mutant obtained by mutating to alanine three hydrophobic residues in the N-terminal domain cannot control the transcription of target genes.

This has led the speculation of a different role played by the N-terminus rather than a direct participation in DNA-binding mechanism: a possible oligomerization domain located in the N-terminal portion of this family of proteins. While Ros87 NMR structural characterization has elucidated the peculiar structural features of the C-terminal portion of Ros protein from *Agrobacterium tumefaciens*, no structural studies concerning the N-terminal region (the first 55 amino acids) were available to date.

The conformational dynamics displayed by the N-domain and the full-length Ros protein concur to suggest a direct role of the C-domain onto the stabilization of the N-term folding. MD simulations analyses return a preferential structural rearrangement of the full-length Ros, whereby the N-domain folds in a two-antiparallel α-helices (namely α′ -Tyr6-Asn27- and α′′ -Glu34-Gly48-) connected to the C-term domain (Ros87), as described above. The N-term domain helices are kept in place and stabilized by hydrophobic interactions at their interfacing surfaces. Significantly, this structural organization is observed in largely more than half of the total full-length Ros simulation time, underscoring the ability of the N-term domain to fold into the two packed antiparallel helices regardless of its initial structuration. On the other hand, in the absence of the C-term domain, MD simulations show that the N-term domain is not capable of adopting a comparable helical rearrangement, yet a smaller 3-helices domain is preferred. Accordingly, the structural characterization of del29-Ros shows how the deletion of the α′ helix (Tyr6-Asn27) disrupts the helical character of the α′′ (Glu34-Gly48) portion. As a result, del29-Ros forms only unstable oligomers (Figs. SI4–SI5), underlining how the hydrophobic interactions established between the two helices (α′ and α′′) are fundamental to obtain a stable tertiary structure capable of promoting oligomerization.

Furthermore, in the full-length Ros simulation, the N-term and C-term domains can rearrange in a more compact globular shape by establishing electrostatic interactions. Nevertheless, the transient character of such contacts (occurrence < 10% of the simulation time), also confirmed by our NMR data, substantiates the hypothesis of a bi-lobal conformation of Ros protein, where the C-term-domain serves/aids the stabilization of the two-helices N-term domain rather than directly lock onto it.

It is worth remarking that the simulations and analyses discussed here have been based on the use of one specific version of the Amber force field^56^. Although it would be preferable to investigate the dynamical features of the modelled proteins described here with different force-fields, it is important to underline that our calculations allowed to capture significant differentiated behaviours of the modelled systems with or without the C-domain. Indeed, our simulations were corroborated by independent experimental evidences endorsing differential roles for the two domains.

Ros shows interesting structural analogies with the Histone-like Nucleoid-Structuring (H-NS) protein that plays a role in the formation of nucleoid structure^53^. Truncations and site-directed mutations proved the importance of the N-terminal domain and of its helical secondary structure to form high order structures also in the case of H-NS^54^. H-NS is made by two functional domains separated by a flexible linker: the N-terminal domain is responsible for the formation of high order structures that bind DNA via the C-terminal domain.

Under this light, the two Ros domains might work as independent units with specific functions: C-term domain harbouring the zinc-finger adopts a conformation that is consistently prone to DNA-binding, regardless of the presence/orientation/interaction of the N-term domain along the entire simulation time whereas N-domain itself might control oligomerization mechanism. Our here reported results convincingly support and integrate our previous findings^23–25^ suggesting that the prokaryotic zinc-finger proteins control genes expression by adopting a mechanism similar to that used by the H-NS proteins found in many other Gram-negative bacteria instead of working as classical eukaryotic transcription factors.

## Methods

### 3D structural models

The full-length Ros protein structural models were produced either by means of comparative modelling^44^ or by estimating amino acid intrinsic secondary structure propensities^45^.

In particular, ab initio modelling of the N-terminal domain (amino acids 1–55) was done using the fully automated server Robetta by Ginzu domain prediction (http://robetta.bakerlab.org). The integrative modeling protocol includes different sequential operations: first, alignments to one or more template structures, by using external programs; then, models based on the template structure are refined by rebuilding the missing parts (loop modelling) and subjected to a full-atom refinement of using the Rosetta full-atom energy function. The final step is a selection of the models using clustering. Sequence alignments and the list of the template proteins used for the modeling are given in Supporting Material (Table SI2).

Protein secondary structure and residue solvent accessibility predictions were also run with Jpred^45^ software generating a fully-extended two helices N-domain (Fig. 1e).

The full-length structural models (142 residues) were built by joining and energy minimizing the so-obtained N-terminal domains with the high-resolution NMR C-terminal domain (Ros87 – 2JSP) using Maestro software^48^ (Fig. 1b–d).

### MD simulations

MD simulations were performed using Gromacs package (v.4.5.5)^55^ with the Amber99sb-ildn force field^56^. All proteins were centered in triclinic boxes allowing a 0.9 nm distance from each box edge and solvated by explicit water molecules (TIP3P model)^57^. Counter-ions were randomly added to neutralize the simulated systems^58^. In details: M1 was solvated by 15111 water molecules; M2 by 7375 water molecules, M3 by 7872 water molecules and all full-length systems were neutralized by 1 Cl^-^. On the other hand, the N-terminal domain (amino acids 1–55) was solvated by 6383 water molecules and neutralized by 6 Na^+^ counter-ions.

Because zinc ion is crucial for protein structure stability, Zn(II) was introduced, tetrahedrally coordinated by Cys79, Cys82, His92 and His97, and distance restraints were applied with a force constant of 1000 kJ/(mol* nm^2^)^59^. Each system was first energy minimized using the steepest descent approach, followed by an equilibration phase where water molecules and protein heavy atoms were position restrained. A short NVT (500 ps) and NPT (500 ns) simulations were run at 300 K previous to the production run. Then the unrestrained systems were kept in an NPT ensemble, at constant temperature of 300 K by the velocity rescaling thermostat^60^ and at a pressure of 1 bar by the Berendsen barostat^61^. Electrostatic interactions were evaluated using the particle mesh Ewald method^62^ and Lennard-Jones forces by a cut-off radius of 0.9 nm. Bond lengths involving hydrogen were restrained by the LINCS algorithm^63^. The time step was set to 2 fs and periodic boundary conditions were applied in all three dimensions. To enhance sampling two independent replicas with different initial velocities were run for each system, totally collecting 0.8 µs of simulation time.

Analyses were carried out on a meta-trajectory produced by concatenating all trajectories of full-length Ros models in order to evaluate conserved dynamic properties of the protein. Four independent 100 ns MD replicas were run for the N-terminal region (amino acids 1–55). Clustering is computed applying the method developed by Daura and collaborators^64^.

### Protein expression and purification

The region of the *ros* gene encoding for the deletion del29-Ros lacking the first 28 amino acids of wild-type Ros, was amplified by PCR using the primer 1 (5′-ACATGCCATGGTCGTTCCGGTAACTGAG-3′), the primer 2 (5′-CGGAATTCTCAACGGTTCGCCTTGC-3′) and the genomic DNA purified from *A. tumefaciens* as a template. The obtained fragment was digested with *NcoI* and *EcoRI* (New England Biolabs) and cloned into a pET-11d vector digested with same restriction enzymes. The sequence of the obtained clone del29-Ros-pET-11d was checked by Sanger method sequencing. del29-Ros-pET-11d was used to transform *E. coli* BL21(DE3) strain to express the protein del29-Ros in M9 salt medium containing ^15^N and ^13^C with the addition of BME vitamin solution (SIGMA). Cell growth was followed by absorbance at 600 nm. The expression was induced with 1 mM IPTG when the bacterial culture reached 0.5 OD_600nm_ and carried out at 28 °C until the culture absorbance at 600 nm reached 1 OD. Then, bacterial cells were harvested by centrifugation and lysed by sonication on ice in presence of cOmplete EDTA-free Protease Inhibitor Cocktail (Roche). The lysate was clarified by centrifugation at 29000 rcf for 30 minutes and total soluble protein fraction was used to purify del29-Ros. The purification was carried out by cation exchange chromatography using a Mono S HR 5/5 column (GE) equilibrated with 20 mM Na_2_HPO_4_ (pH 6.8) and by a following step using a HiLoad 26/60 Superdex 75 gel filtration chromatography column (GE) equilibrated with a buffer composed by 20 mM Na_2_HPO_4_ (pH 6.8) and 0.2 M NaCl. It should be here noted that, after the first step of purification, we were not able to obtain good quality spectra as the solution obtained appeared as a mixture of different oligomeric species (data not shown); only the second step (gel filtration) allowed the separation of a monomeric soluble form of del29-Ros.

### Molecular weight determination by Light scattering

For molecular weight measurements, a MiniDAWN Treos spectrometer (Wyatt Instrument Technology Corp.) was connected on-line to a size-exclusion chromatography. A sample of 1 mg was loaded on a Superdex 75 (10×30 cm, GE-Healthcare) equilibrated in the same buffer used for purification procedures and connected to a triple-angle light scattering detector equipped with a QELS (Quasi- Elastic Light Scattering) module. A constant flow rate of 0.5 ml/min was applied. Elution profiles were detected by a Shodex interferometric refractometer. Data were analyzed by using Astra 5.3.4.14 software (Wyatt Technology).

### NMR

All NMR samples contained 200 µM of ^15^N-^13^C double-labelled del29-Ros protein in 20 mM phosphate buffer, 0.2 M NaCl, pH = 6.8, with 10% of D_2_O. The experiments were acquired at 298 K on the Bruker Avance III HD 600 MHz equipped with cryoprobe at the Environmental, Biological and Pharmaceutical Sciences and Technologies Department of the University of Campania “Luigi Vanvitelli” (Caserta, Italy). To allow sequence-specific backbone and Cβ resonances assignment, bidimensional ^1^H-^15^N HSQC and a standard set of triple-resonance NMR experiments were acquired (i.e. 3D HNCA^65^, 3D HNCO^66^, 3D CBCANH^65^, and 3D CBCA(CO)NH^65^). The translational diffusion coefficient (D_t_) was obtained by pulsed field gradient spin-echo DOSY experiments^67,68^. All the spectra were processed using the software TOPSPIN 4.0 (Bruker) and analyzed with the software CARA^69^. ^1^H − ^15^N HSQC J-18 spectra were acquired using as transfer delays a τ_m_ [1/(4 J)] of 13.8 ms, to obtain the coherence transfer from the H_ε1_ and H_δ2_ histidine side chain protons to N_ε2_ and N_δ1_ through the ^2^J_HN_ coupling constant^70–73^.

HN and ^15^N chemical shift perturbations were estimated by using the following equation:^74^

$${\Delta }_{av}(HN)=\sqrt[2]{\frac{\Delta {H}^{2}+{\left(\frac{\Delta N}{5}\right)}^{2}}{2}}$$

where ΔHN and ΔN are the differences between the chemical shifts of Ros87^30^ and del29-Ros.

### Chemical cross-linking of Ros87 and del29-Ros

100 μM of Ros87 or del29-Ros were incubated with dimethyl adipimidate (DMA) in NaP 20 mM at room temperature for 0,5 and 1 hour in a molar ratio of 1:10 and 1:20. The reactions were stopped with Tris/HCl 50 mM pH 8.0. The reactions were analyzed on 15% SDS-PAGE.

## Supplementary information

Supplementary information.
