# Supplementary material for: Structural Insight of the Full-Length Ros Protein: A Prototype of the Prokaryotic Zinc-Finger Family
Source: Sci Rep. 2020 Jun 9;10:9283. doi: 10.1038/s41598-020-66204-5 (PMC7283297; doi:10.1038/s41598-020-66204-5)
Supplement: Supplementary file 1 — Supplementary information. [file 41598_2020_66204_MOESM1_ESM.pdf]

STRUCTURAL INSIGHT OF THE FULL-LENGTH ROS PROTEIN: A PROTOTYPE OF THE PROKARYOTIC ZINC-FINGER FAMILY.

D'Abrosca Gianluca[1#], Paladino Antonella[1,2#], Baglivo Ilaria[1], Russo Luigi[1], Sassano Marica[1], Grazioso Rinaldo[1], Iacovino Rosa[1], Pirone Luciano[3], Pedone Emilia Maria[3], Pedone Paolo Vincenzo[1], Isernia Carla[1], Fattorusso Roberto[1], Malgieri Gaetano[1*]

[1] Department of Environmental, Biological and Pharmaceutical Sciences and Technologies, University of Campania "Luigi Vanvitelli", via Vivaldi, 43 – 81100 Caserta, Italy.

[2] SCITEC-CNR, via Mario Bianco 9, 20131 Milano, Italy.

[3] Institute of Biostructures and Bioimaging - CNR, Via Mezzocannone 16, 80134 Naples (Italy).

[#] these authors equally contributed to the work.

SUPPLEMENTARY INFORMATION

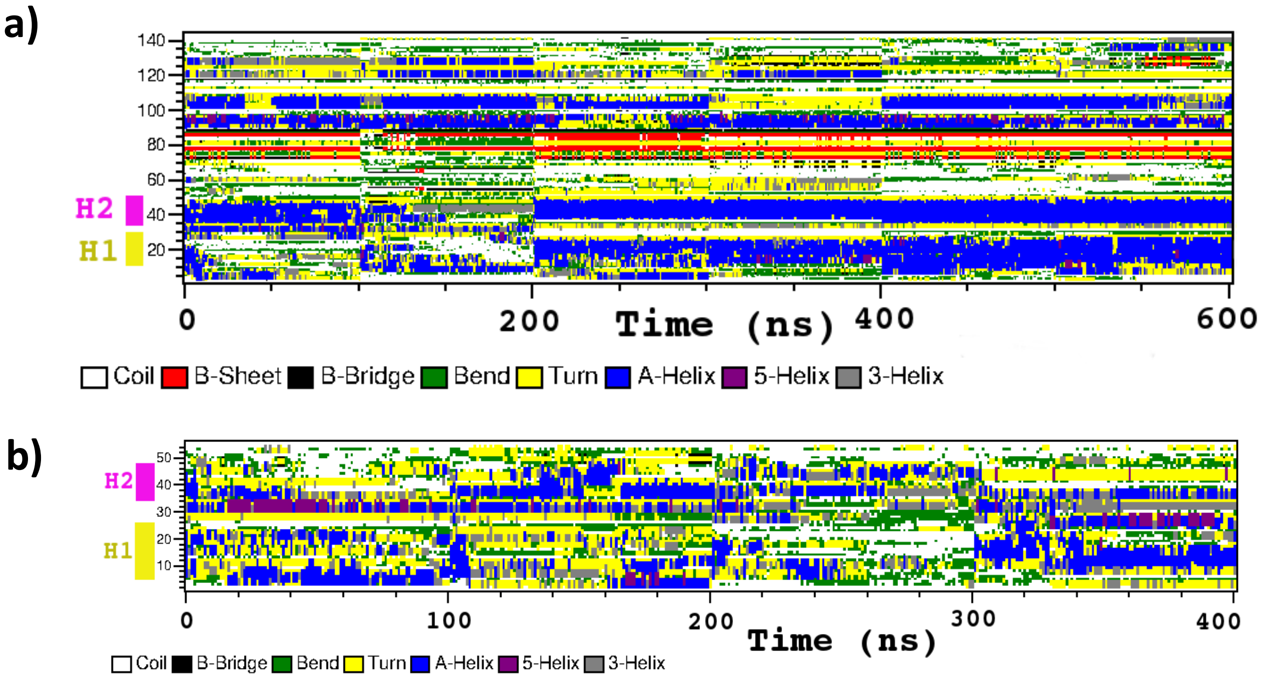

Figure SI1. *Time evolution of the Secondary Structure Elements. a) N-domain (aa 1-55) and C-domain (amino acids 56-142) are highlighted on the y-axis along the full meta-trajectory (600 ns) of the full-length protein simulation. b) N-term domain (amino acids 1 to 55) simulation. H1 corresponds to helix 6-27 and H2 to helix 34-48. See main text.*

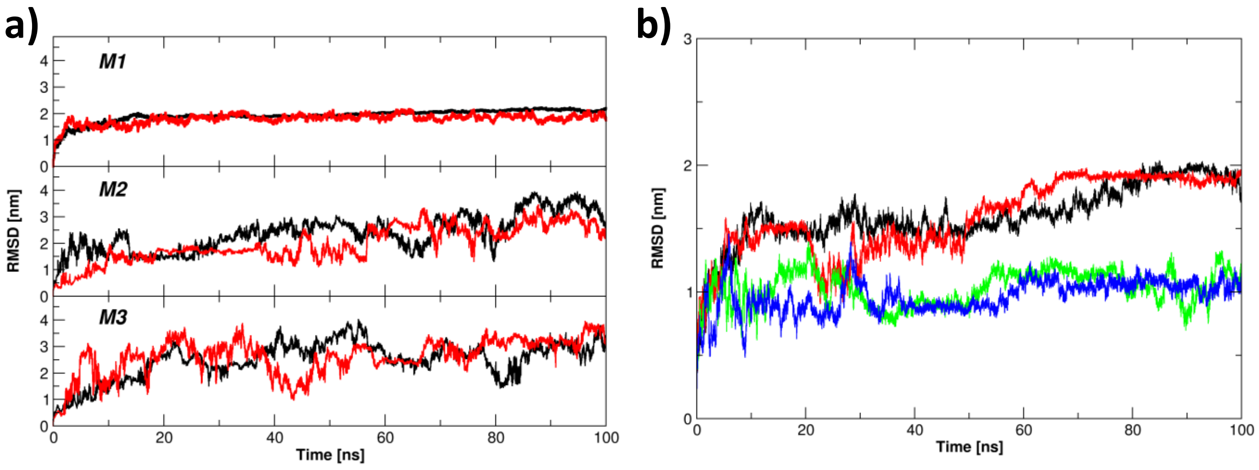

Figure SI2. *RMSD calculations. Root mean square deviations on c-alpha atoms along simulation time are reported for full-length models in a) and the N-domain in b). Calculations are M1-M3 are labelled and different replicas are shown in different colors.*

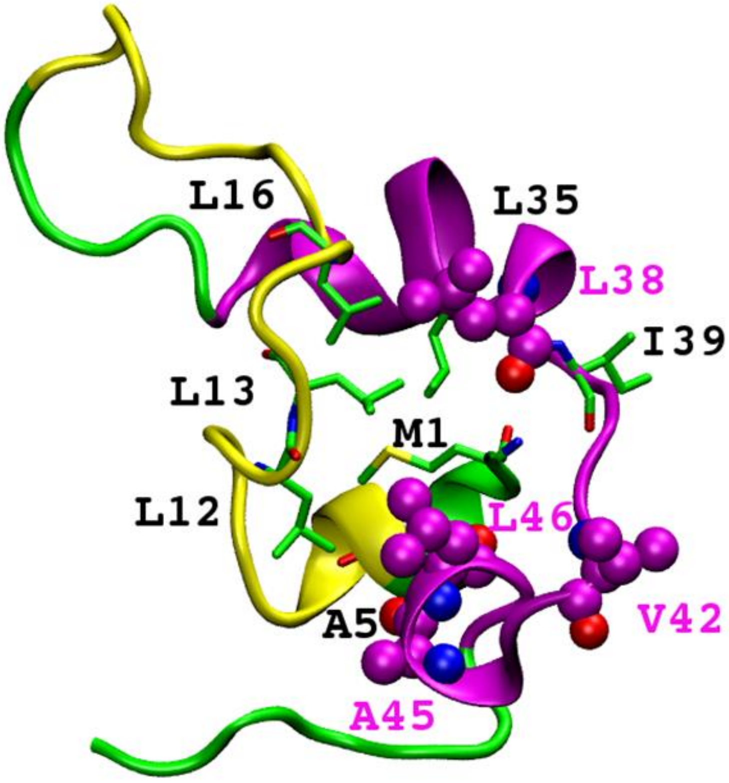

Figure SI3. *Hydrophobic assembly of the N-terminal domain (cluster 3). Hydrophobic amino acids from the N-domain α" helix (according to figure SI1), and residues within a 5 Å shell are labeled and evidenced in CPK and sticks, respectively. Helix α' (Y6-N27) is shown in yellow cartoons and helix α" (E34-G48) in magenta.*

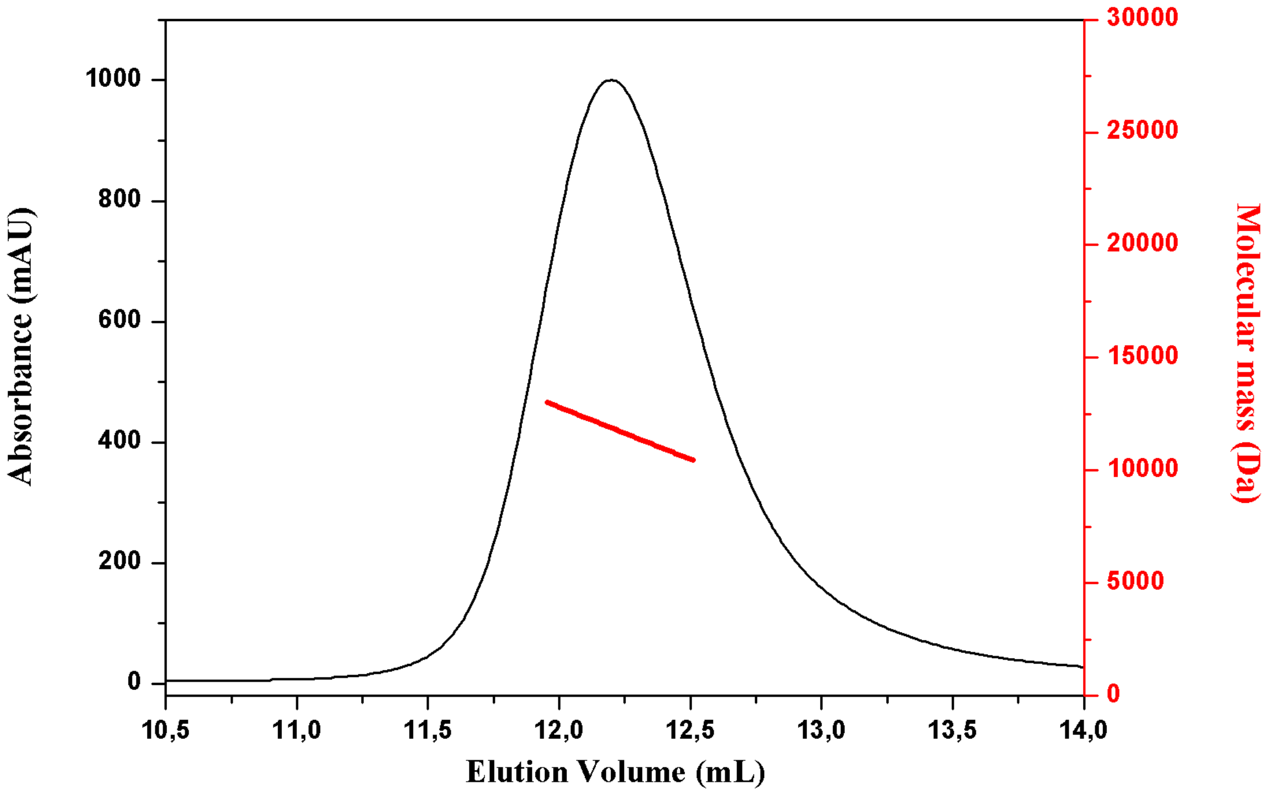

Figure SI4*. Light-scattering measurement of del29-Ros. The plot reports the molecular mass (red line) and absorbance at 280 nm (black line) versus the elution volume*

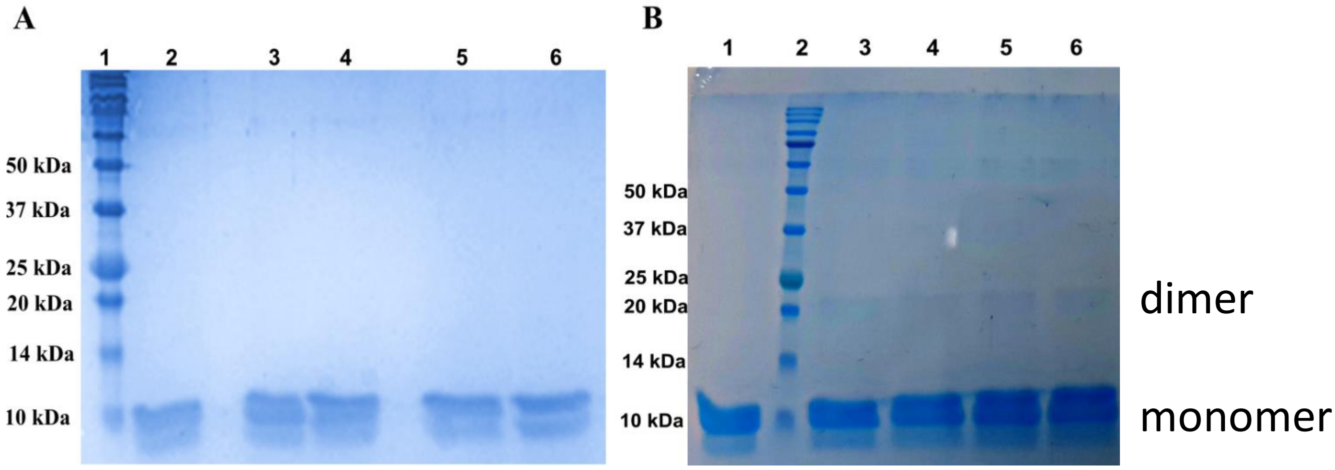

Figure SI5. *Chemical cross-linking of Ros87 (A) and del29-Ros (B) with DMA. 100 μM of proteins were incubated with and without the cross-linker and analyzed on 15% SDS-PAGE. Ros87 in A) lane 1: molecular weight markers, lane 2: Purified recombinant Ros87; lanes 3-6 incubated for 0.5 and 1 hour in the presence of DMA at a molar ratio of 1:10 (lane 3-4) and 1:20 (5-6). del29-Ros in B) lane 1 Purified recombinant del29-Ros, lane 2: molecular weight markers, lanes 3-6 incubated for 0.5 and 1 hour in the presence of DMA at a molar ratio of 1:10 (lane 3-4) and 1:20 (5-6). Positions monomers and dimers are indicated.*

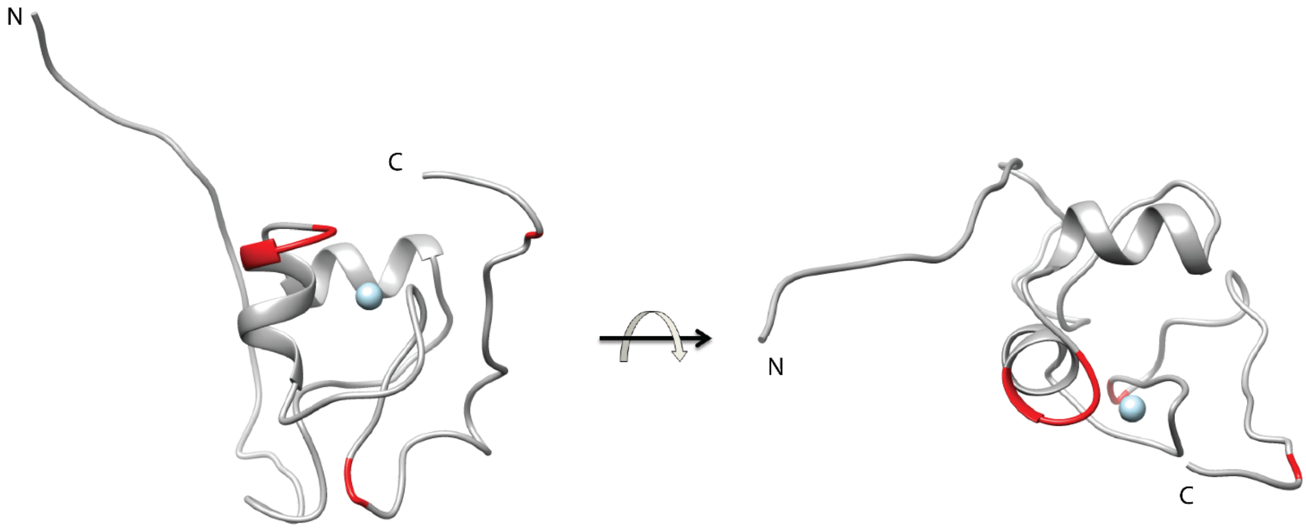

Figure SI6. *Chemical Shift Mapping. Mapping onto the Ros87 NMR structure (PDB code 2JSP) of the Chemical Shift Perturbations reported in Figure 5e.*

#NUM	AA	CA	CB	CO	N	HN
30	V	-	-	-	-	-
31	P	56.87	-	176.86	-	-
32	V	62.72	32.31	176.47	120.58	8.21
33	T	61.79	69.49	174.21	117.11	8.03
34	E	55.94	30.16	175.76	123.39	8.25
35	L	52.71	41.40	-	124.77	8.16
36	P	63.18	31.77	177.47	-	-
37	G	45.01	-	173.91	108.62	8.31
38	L	55.10	42.24	177.36	121.52	7.96
39	I	60.89	38.23	176.16	122.02	8.11
40	S	58.10	63.72	-	119.53	8.24
41	D	54.10	41.02	176.16	122.75	8.27
42	V	62.26	32.24	-	119.75	7.95
43	H	55.56	29.08	-	-	-
44	T	61.87	69.49	174.22	115.75	8.04
45	A	52.33	18.84	177.67	126.43	8.29
46	L	55.02	41.94	177.57	121.27	8.17
47	S	58.18	63.64	175.03	116.18	8.17
48	G	45.40	-	174.41	110.75	8.36
49	T	61.64	69.57	174.60	113.21	8.02
50	S	58.18	63.64	173.62	118.15	8.25
51	A	50.48	18.07	-	127.03	8.22
52	P	57.26	30.32	-	-	-
53	A	52.25	19.05	177.86	118.14	8.34
54	S	58.10	63.64	174.43	114.86	8.19
55	V	61.85	32.75	175.64	121.36	7.99
56	A	52.25	19.15	177.45	127.74	8.24
57	V	61.78	32.70	175.74	119.23	8.01
58	N	52.79	38.78	175.03	122.48	8.42
59	V	62.24	32.37	176.21	120.72	8.06
60	E	55.48	29.16	-	121.87	8.50
61	K	-	-	-	-	-
62	Q	-	-	-	-	-
63	K	-	-	-	-	-
64	P	-	-	-	-	-
65	A	54.09	18.74	177.57	122.84	8.39
66	V	59.41	34.62	173.62	111.27	7.35
67	S	57.74	63.56	176.31	115.63	8.01
68	V	66.34	31.78	179.92	124.89	8.64
69	R	57.87	29.70	176.86	120.89	8.47
70	K	55.56	32.31	176.86	118.90	7.72
71	S	60.49	63.64	172.62	114.67	7.83
72	V	62.72	32.31	175.41	121.21	7.77
73	Q	53.17	29.62	174.72	125.75	8.08
74	D	57.71	40.47	175.97	117.70	8.30
75	D	52.56	41.40	175.84	110.96	8.01

76	H	55.94	30.08	183.05	112.69	7.19
77	I	59.18	41.40	175.05	120.08	9.24
78	V	60.92	32.78	175.60	127.26	9.09
79	C	59.53	30.53	177.47	129.15	9.05
80	L	56.79	39.14	178.10	131.00	9.28
81	E	58.18	29.62	177.26	117.40	9.00
82	C	58.10	32.78	176.36	112.64	7.86
83	G	45.86	-	173.93	114.23	8.52
84	G	44.71	-	170.86	110.30	8.52
85	S	57.33	64.49	173.31	115.52	7.57
86	F	57.25	43.63	175.03	120.67	8.85
87	K	59.10	32.31	-	122.21	8.85
88	S	55.39	63.34	175.52	108.84	7.59
89	L	55.94	44.01	177.97	127.30	8.76
90	K	61.49	32.31	-	117.95	7.99
91	R	-	-	-	-	-
92	H	-	27.31	176.55	-	-
93	L	58.63	41.47	180.21	118.01	8.62
94	T	65.87	68.95	176.76	113.23	7.60
95	T	64.49	69.34	-	112.57	8.16
96	H	57.16	30.33	-	118.19	8.36
97	H	54.08	30.09	173.21	111.66	6.67
98	S	58.34	61.79	173.31	113.98	7.39
99	M	54.10	38.70	174.84	117.53	7.66
100	T	59.10	68.64	-	111.40	8.38
101	P	65.87	32.31	177.98	-	-
102	E	60.41	28.70	179.50	115.72	8.77
103	E	58.64	30.08	176.34	120.76	7.82
104	Y	61.79	39.63	176.57	121.96	8.26
105	R	60.02	29.85	178.71	116.17	8.67
106	E	58.64	29.55	179.19	116.86	7.76
107	K	59.08	32.31	177.88	120.46	7.98
108	W	55.56	28.77	173.59	115.25	7.14
109	D	54.95	38.69	175.03	119.14	7.54
110	L	51.83	40.51	177.69	117.94	8.55
111	P	62.26	32.78	177.69	-	-
112	V	64.57	31.45	-	117.86	8.43
113	D	52.06	39.63	176.07	115.79	7.99
114	Y	56.79	39.55	-	124.97	7.87
115	P	63.26	32.31	174.02	-	-
116	M	63.18	35.47	176.35	111.46	8.06
117	V	60.02	35.09	174.41	118.99	7.45
118	A	50.94	16.85	-	129.72	8.62
119	P	65.72	31.85	178.17	-	-
120	A	54.10	18.23	178.54	117.94	8.58
121	Y	57.71	38.70	175.85	116.29	7.83
122	A	53.17	18.84	178.60	123.82	7.70

123	E	57.25	29.70	176.97	119.53	8.29
124	A	53.25	18.38	178.41	123.62	8.06
125	R	56.87	30.08	177.07	118.60	8.11
126	S	59.10	63.26	-	115.94	8.07
127	R	57.31	30.33	-	122.40	8.13
128	L	55.84	41.86	177.59	121.27	7.95
129	A	53.17	18.77	-	123.27	7.98
130	K	56.95	32.70	-	119.37	8.01
131	E	-	-	-	-	-
132	M	55.87	32.70	176.97	-	-
133	G	45.48	-	174.43	109.08	8.24
134	L	55.48	41.94	178.19	121.32	8.07
135	G	45.48	-	174.38	109.08	8.43
136	Q	55.94	29.24	175.45	119.54	8.09
137	R	53.94	32.31	-	125.63	8.56
138	R	-	-	-	-	-
139	K	56.33	32.85	176.14	-	-
140	A	52.25	19.15	176.21	125.08	8.27
141	N	53.17	38.78	174.12	118.25	8.35
142	R	57.25	31.39	-	125.68	7.79

Table SI1. *Chemical Shift list of* ^{1}H, ^{15}N *and* ^{13}C *resonances of del29-Ros.*

```
## 76145 3zkvA_101
# filt alignment (0.34% id)
scores_from_program: 39.37 0.032
8 -------------------------------------------------------------------------------------
-------------------------------AQDLLVELTADIVAAYVSNHVVPVTELPGLISDVHTALSGTSAP------------
---------------------------------------------------------------------------------------
---------------------------------------------------------------------------------------
---------------------------------------------------------------------------------------
---------------------------------------------------------------------------------------
---------------------------------------------------------------------------------------
---------------------------------------------------------------------------------------
---------------------------------------------------------------------------------------
------------------------------------------------------------------------
0
QEVQFFGAITLHSKLMKHWHEVPKIVLNRLCISLGAYIVHMLGGAIEEVINTFQNQRMSADVQLWIMLEVLTAIPEEAQVIHTSVKRVV
LRAEIAKRVQLVIHTVERYLKLQMNRVWDAEAYSNMNRAVKCVGTWIKNIGYTIEGCVTITAVLLEVVHKCYWPCIHGCMTADENELAE
SCLKTMVNIIIQPDCHNYPKTAFVLIKMFLDSLSEITKTEWKRENDNEDIIVHIYMLFVSSVERHSTLLLSGITSADPELSILVHRIVQ
EILHCTDKPGIYPVEESCSTMALAFWYMLQDEVFAMSDEQKHKCWEYIKPLYAHLTRILVRKSEQPDEKSLAKWSSDDLECFRCYRQDI
SDTFMYCYDVLNDYILEILAAMLDEAIADLQRHPTHWTKLEACIYSFQSVAEHFGGEEKRQIPRLMRVLAEIPYEKLNVKLLGTALETM
GSYCNWLMENPAYIPPAINLLVRGLNSSMSAQATLGLKELCRDCQLQLKPYADPLLNACHASLNTGRMKNSDSVRLMFSIGKLMSLLRP
EEIPKYLDIIVSPCFEELQAICQADSKTPAARIRTIFRLNMISTLFSSLNTDVDDQPIVQPVLLVMQRTMPIFKRIAEMWVEEIDVLEA
ACSAMKHAITNLRSSFQPMLQDLCLFIVASFQCAPTLEISKTAIVMFFKDEGCKPLMQQLLREFIQHSFKLFESTPEQNFSNISDTMET
FFGCLTQIIKKIPQVLEDKTLAYDRLVFYAQRGMTLPESGAIRNSIQFLTHFVMQSRNHAHVTEVVLATGEQTLYTAMMCVGYLTPRSQ
VDKFADILLAMNRKYAAEMAVWMKSLMSTPNFPTQLITDADKTRYTALIIKENKRLLQQHLSEMAMKTRGLT
--
## 76145 2x1gF_102
# filt alignment (0.33% id)
scores_from_program: 39.04 0.034
8 -------------------------------------------------------------------------------------
---------------------------------------------------------------------------------------
```

AQDLLVELTADIVAAYVSNHVVPVTELPGLISDVHTALSGTSAP---

0
EAVVSFYRSNSQNHEWLTDAEASPQAWQFSWQLMQLGKSQEVQFFGAITLHSKLMKHWHEVPPENREELKQKILESIVRFAGGPKIVLN
RLCISLGAYIVHMLGEEVINTFQNQRSADVQLWIMLEVLTAIPEEAQVIHTSVKRVVLRAEIAKRVQLVIHTVERYLKLQMNRVWDAEA
YSNMNRAVKCVGTWIKNIGYTIEGCVTITAVLLEVVHKCYWPCIHGCMTADENELAESCLKTMVNIIIQPDCHNYPKTAFVLIKMFLDS
LSEITKTEWKRENDNEDIIVHIYMLFVSSVERHSTLLLSGITSADPELSILVHRIVQEILHCTDKPGIYPVEESCSTMALAFWYMLQDE
VFAHKCWEYIKPLYAHLTRILVRKSEQPDEKSLAKWSSDDLECFRCYRQDISDTFMYCYDVLNDYILEILAAMLDEAIADLQRHPTHWT
KLEACIYSFQSVAEHRQIPRLMRVLAEIPYEKLNVKLLGTALETMGSYCNWLMYIPPAINLLVRGLNSSMSAQATLGLKELCRDCQLQL
KPYADPLLNACHASLNTGRMKNSDSVRLMFSIGKLMSLLRPEEIPKYLDIIVSPCFEELQAICQATPAARIRTIFRLNMISTLFSSLNT
PVLLVMQRTMPIFKRIAEMWVEEIDVLEAACSAMKHAITNLRSQPMLQDLCLFIVASFQCCAPTLEISKTAIVMFFKPLMQQLLREFIQ
HSFKLFESTPEQNFSNISDTMETFFGCLTQIIKKIPQVLEDKTLAYDRLVFYAQRGMTLPESGAIRNSIQFLTHFVMQSRNHAHVTEVV
LATGEQTLYTAMMCVGYLTPRSQVDKFADILLAMNRKYAAEMAVWMKSLMSTPNFPTQLITDADKTRYTALIIKEKVNKRLLQQHLSEM
AMKTRG
--
76145 5hiuA_103
filt alignment (0.98% id)
scores_from_program: 38.47 0.036
8 ---

--------------AQDLLVELTADIVAAYVSNHVVPVTELPGLISDVHTALSGTSAP-------------------------------
----

0
SPEQADLVAKLKNGHLSERVLAANKLRFAVVDFPLNPVHAIWHAAKDMIHPENPDNARQASWELLIECVKYPNSTELERSEYFHTLTGP
AHSKDFCYQLVALEQLTNHGRNIAGFYYEMFPLLTLWLNQAYRAARDARKLALARPASPEDKNLSQLFALVKDVIKFNFKFATDDVIAG
LIDMLLKICMLTSVEDDLRACIHVIESLVTFGSIPTNKLKYCIQVLSSIHCLVPSLQKEAWHTISIICRSHHGQSTVRILLDFLRSYSP
NPDKNREKDTVRDVRGALSVLQKLLRKTAEKGYPQVPLSLLVGGLANVSKSSSTRVATEILRLINSLFHGNINPILVEEHWEPIFDVAA
QCATKAPTVAKENVSLQLKHLILRVENLIVHQGPELLQRDDCMKFLIRVQH
--
76145 5hiuB_104
filt alignment (0.98% id)
scores_from_program: 38.26 0.037
8 ---

-------------AQDLLVELTADIVAAYVSNHVVPVTELPGLISDVHTALSGTSAP--------------------------------
----

0
SPEQADLVAKLKNGHLSERVLAANKLRFAVVDFPLNPVHAIWHAAKDMIHPENPDNARQASWELLIECVKYPNSTELERSEYFHTLTGP
AHSKDFCYQLVALEQLTNHGRNIAGFYYEMFPLLTLWLNQAYRAARDARKLALAPASPEDKNLSQLFALVKDVIKFNFKFATDDVIAGL
IDMLLKICMLTSVEDDLRACIHVIESLVTFGSIPTNKLKYCIQVLSSIHCLVPSLQKEAWHTISIICRSHHGQSTVRILLDFLRSYSPN
PDKNREKDTVRDVRGALSVLQKLLRKTAEKGYPQVPLSLLVGGLANVSKSSSTRVATEILRLINSLFHGNINPILVEEHWEPIFDVAAQ
CATKALPTVAKENVSLQLKHLILRVENLIVHQGPELLQRDDCMKFLIRVQH
--
76145 5vchB_105
filt alignment (0.39% id)
scores_from_program: 37.58 0.04
8 ---

--AQDLLVELTADIVAAYVSNHV--
VPVTELPGLISDVHTALSGTSAP--

-----------------------!

0
LPALIHILQNSSNDGIKQLAGVEARKQVSKHWGSLDAATQTSVKQSLLNSAFNEGKDAVRHANARVIASIGSEELDEKKWPELIPNLLQ
AACDSNPKIRETAIFIILSLLESFNANLALHIDDFLNLFAQTINDSASLETRSLSAQALSYVSSLIEEEGEINPQYAAKFASLIPSVVQ
VLDATIREGDTTNTKLIFNCLNDFLLLDSQLTGNTIADLVKLALQIAVNSDVDEDIRVFAVQFVTSALVYRKSKINQAKLGPEITLAAL
KVASEEIDVEDELTNEDEENTPALTALRLISNASGELSPSQVGVPIIEHLPTMLSSSNPFERRSILLAISVLVTGSPDYTLSQFDKIIP
ATVTGLKDSEAVVQLAALKCIVQLSTNLQDEVARYHEQYLPLVIDIIDSAKHVVIYKYATLALDGLLEFIAHNDIIKYLDPLMNKLFQM
LETQQSPKLRAAIVSAIGSCAFAAGSGFVPYFKTSVQYLQQFIQNVSQIEGLSEDDIELKALTFENISTMGRAVKSAAFAEYAEPLVNA
AYEAIKTDSARLRESGYAFIANMAKVYGKDFAPFLQTIIPEIFKTLEQEEYTVNTGIAYEKEVAAAALSELAIASKEHFLEYVEPSLKV
LAEQVNESYGLKETALHSMWAIVKAVLLTANLKEGEYPKGVPSGSYVDASALAVIQTVREVSLNNVIEEVETSMVISVFQDLSEMLRLF
GPIIIMDNGDSTHLDQLCREALSVLKGEHACQTIDASETEATLLDVALDIYVALSTNLVGGFAQVFTTAKPVILQLCQSKSKNKRSFAV
GALSEIALGMRDENPFIQELLEALIISLTNDKSLEVRCNASYGVGLLIEYSSFDVSAIYSPVLKSLYEILSVADEKNLDDEATKEIVDR
TFSNVCGCVARMILKHQNLVPLEHTIPALLSHLPFNTAFEEYDPIFKLFLKLFQEQNSTIINEAPKVIAIFATVFEKESERIELETNST
LGREENLE!
 KRKQFQSEEIKQQVIELLKHLNQQFNGAVAQNPVLAQVIA
--
76145 5ve8B_106
filt alignment (0.39% id)
scores_from_program: 37.57 0.04
8 ---

---AQDLLVELTADIVAAYVSNHV--
VPVTELPGLISDVHTALSGTSAP--

----------------------!

0
ALPALIHILQNSSNDGIKQLAGVEARKQVSKHWGSLDAATQTSVKQSLLNSAFNEGKDAVRHANARVIASIGSEELDEKKWPELIPNLL
QAACDSNPKIRETAIFIILSLLESFNANLALHIDDFLNLFAQTINDSASLETRSLSAQALSYVSSLIEEEGEINPQYAAKFASLIPSVV
QVLDATIREGDTTNTKLIFNCLNDFLLLDSQLTGNTIADLVKLALQIAVNSDVDEDIRVFAVQFVTSALVYRKSKINQAKLGPEITLAA
LKVASEEIDVEDELTNEDEENTPALTALRLISNASGELSPSQVGVPIIEHLPTMLSSSNPFERRSILLAISVLVTGSPDYTLSQFDKII
PATVTGLKDSEAVVQLAALKCIVQLSTNLQDEVARYHEQYLPLVIDIIDSAKHVVIYKYATLALDGLLEFIAHNDIIKYLDPLMNKLFQ
MLETQQSPKLRAAIVSAIGSCAFAAGSGFVPYFKTSVQYLQQFIQNVSQIEGLSEDDIELKALTFENISTMGRAVKSAAFAEYAEPLVN
AAYEAIKTDSARLRESGYAFIANMAKVYGKDFAPFLQTIIPEIFKTLEQEEYQFTVNTGIAYEKEVAAAALSELAIASKEHFLEYVEPS
LKVLAEQVNESYGLKETALHSMWAIVKAVLLTANLKEGEYPKGVPSGSYVDASALAVIQTVREVSLNNVIEEVETSMVISVFQDLSEML
RLFGPIIIMDNGDSTHLDQLCREALSVLKGEHACQTIDLDASETEATLLDVALDIYVALSTNLVGGFAQVFTTAKPVILQLCQSKSKNK
RSFAVGALSEIALGMRDENPFIQELLEALIISLTNDKSLEVRCNASYGVGLLIEYSSFDVSAIYSPVLKSLYEILSVADEKNDEATKEI
VDRTFSNVCGCVARMILKHQNLVPLEHTIPALLSHLPFNTAFEEYDPIFKLFLKLFQEQNSTIINEAPKVIAIFATVFEKESERIELET
NSTLGREE!
 NLEKRKQFQSEEIKQQVIELLKHLNQQFNGAVAQNPVLAQVIA
--
76145 5hiuD_107
filt alignment (1.00% id)
scores_from_program: 37.56 0.04
8 ---

-----------AQDLLVELTADIVAAYVSNHVVPVTELPGLISDVHTALSGTSAP----------------------------------
----

0
SPEQADLVAKLKNGHLSERVLAANKLRFAVVDFPLNPVHAIWHAAKDMIHPENPDNARQASWELLIECVKYPNSTELERSEYFHTLTGP
AHSKDFCYQLVALEQLTNHGRNIAGFYYEMFPLLTLWLNQAYRAARDARKLAPASPEDKNLSQLFALVKDVIKFNFKFATDDVIAGLID
MLLKICMLTSVEDDLRACIHVIESLVTFGSIPTNKLKYCIQVLSSIHCLVPSLQKEAWHTISIICRSHHGQSTVRILLDFLRSYKDTVR
DVRGALSVLQKLLRKTAEKGYPQVPLSLLVGGLANVSKSSSTRVATEILRLINSLFHGNINPILVEEHWEPIFDVAAQCATKAVTLPLP
TVAKEEPVVEDNVSLQLKHLILRVENLIVLLQRDDCMKFLIRVQH

```
--
## 76145 5bk46_108
# filt alignment (0.16% id)
scores_from_program: 37.41 0.041
12 --------------------------------------------------------------------------------------
-----------------------------------------------------------------------------------------
-----------------------------------------------------------------------------------------
-----------------------------------------------------------------------------------------
-----------------------------------------------------------------------------------------
-----------------------------------------------------------------------------------------
---------------------------------------------LVELTADIVAAYVSNHVVPVTELPGLISDVHTALSGTSA
0
VDDVTGEKVREAFEQFLEDFSVQSTDTGEVEKVYRAQIEFMKIYDLNTIYIDYQHLSMRENGALAMAISEQYYRFLPFLQKGLRRVVRK
YAPERVFQISFFNLPTVHRIRDIRSEKIGSLLSISGTVTRTSEVRPELYKASFTCDMCRAIVDNVEQSFKYTEPTFCPNPSCENRAFWT
LNVTRSRFLDWQKVRIQENANEIPTGSMPRTLDVILRGDSVERAKPGDRCKFTGVEIVVPDVTQLGLPGVKPSSTLDTRGISKTSLGVR
DLTYKISFLACHVISIGSDEINELKEMVKDEHIYDKLVRSIAPAVFGHEAVKKGILLQMLGGVHKSTVEGIKLRGDINICVVGDPSTSK
SQFLKYVVGFAPRSVYTSGKASSAAGLTAAVVRDEEGGDYTIEAGALMLADNGICCIDEFDKMDISDQVAIHEAMEQQTISIAKAGIHA
TLNARTSILAAANPVGGRYNRKLSLRGNLNMTAPIMSRFDLFFVILDDCNEKIDTELASHIVDLHMKRDEAIEPPFSAEQLRRYIKYAR
TFKPILTKEARSYLVEKYKELRKDDAQGFSRSSYRITVRQLESMIRLSEAIARANCVDEITPSFIAEAYDLLRQSIIRVDV
--
## 76145 5hiuC_109
# filt alignment (1.00% id)
scores_from_program: 37.4 0.041
8 ---------------------------------------------------------------------------------------
-----------------------------------------------------------------------------------------
-----------AQDLLVELTADIVAAYVSNHVVPVTELPGLISDVHTALSGTSAP----------------------------------
-----------------------------------------------------------------------------------------
------------------------------------------------
0
SPEQADLVAKLKNGHLSERVLAANKLRFAVVDFPLNPVHAIWHAAKDMIHPENPDNARQASWELLIECVKYPNSTELERSEYFHTLTGP
AHSKDFCYQLVALEQLTNHGRNIAGFYYEMFPLLTLWLNQAYRAARDARKLAPASPEDKNLSQLFALVKDVIKFNFKFATDDVIAGLID
MLLKICMLTSVEDDLRACIHVIESLVTFGSIPTNKLKYCIQVLSSIHCLVPSLQKEAWHTISIICRSHHGQSTVRILLDFLRSYKDTVR
DVRGALSVLQKLLRKTAEKGYPQVPLSLLVGGLANVSKSSSTRVATEILRLINSLFHGNINPILVEEHWEPIFDVAAQCATKAVTLPLP
TVAKEEPVVEDNVSLQLKHLILRVENLIVHLLQRDDCMKFLIRVQH
--
## 76145 1ao6A_110
# filt alignment (1.21% id)
scores_from_program: 37.29 0.042
11 -------------LLVELTADIVAAYVSNHVVPVTELPGLISDVHTALSGTSAP--------------------------------
-----------------------------------------------------------------------------------------
-----------------------------------------------------------------------------------------
-----------------------------------------------------------------------------------------
-----------------------------------------------------------------------------------------
-----------------------------------------------------------------------------------------
-------------------------------------------------
0 SEVAHRFKDLGEENFKALVLIAFAQYLQQC--
PFEDHVKLVNEVTEFAKTCVADESAENCDKSLHTLFGDKLCTVATLRETYGEMADCCAKQEPERNECFLQHKDDNPNLPRLVRPEVDVM
CTAFHDNEETFLKKYLYEIARRHPYFYAPELLFFAKRYKAAFTECCQAADKAACLLPKLDELRDEGKASSAKQRLKCASLQKFGERAFK
AWAVARLSQRFPKAEFAEVSKLVTDLTKVHTECCHGDLLECADDRADLAKYICENQDSISSKLKECCEKPLLEKSHCIAEVENDEMPAD
LPSLAADFVESKDVCKNYAEAKDVFLGMFLYEYARRHPDYSVVLLLRLAKTYETTLEKCCAAADPHECYAKVFDEFKPLVEEPQNLIKQ
NCELFEQLGEYKFQNALLVRYTKKVPQVSTPTLVEVSRNLGKVGSKCCKHPEAKRMPCAEDYLSVVLNQLCVLHEKTPVSDRVTKCCTE
SLVNRRPCFSALEVDETYVPKEFNAETFTFHADICTLSEKERQIKKQTALVELVKHKPKATKEQLKAVMDDFAAFVEKCCKADDKETCF
AEEGKKLVAASQAA
--
```

***Table SI2**. ROBETTA prediction: alignments used for comparative modeling.*
